# Supplementary material for: Female and Combined Male–Female Injury Risk Functions for the Anterior Pelvis Under Frontal Lap Belt Loading Conditions
Source: Ann Biomed Eng. 2025 Jul 10;53(10):2669–78. doi: 10.1007/s10439-025-03777-0 (PMC12457498; doi:10.1007/s10439-025-03777-0)

Supplementary Material for

Hanggi C, Kong J, Caldwell J, Gepner B, Östling M, Kerrigan J. *Female and Combined Male-Female Injury Risk Functions for the Anterior Pelvis Under Frontal Lap Belt Loading Conditions*. Annals of Biomedical Engineering. (2025).

Contents

Figure A1 2
Figure A2 2
Figure A3 3
Figure A4 4
Table A1 5
Figure A5 6
Figure A6 7

Figure A1: 3D reconstructions of pelvis fractures from PMHS sled tests [6].

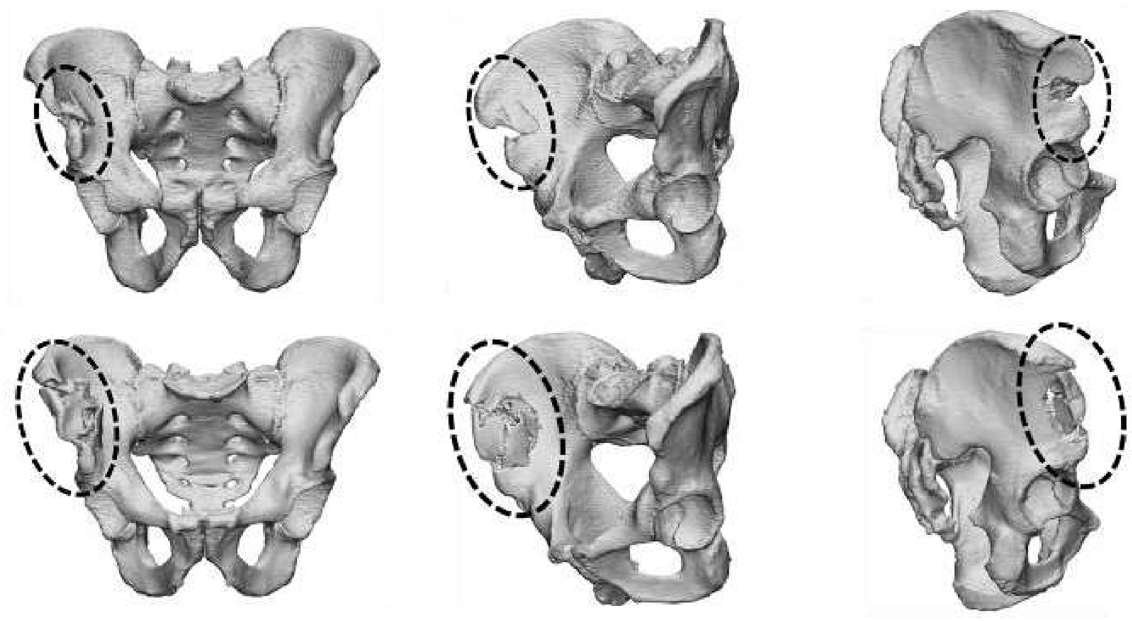

Figure A2: Lateral view of iliac wing depicting the Notch to Belt Angle, which was used as a guide for belt orientation in [13-14] and the current study.

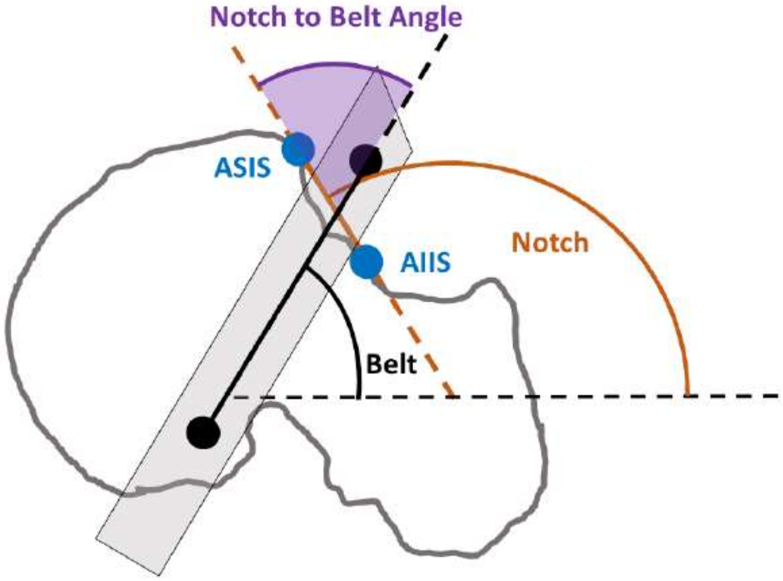

Figure A3: a) lateral/medial and b) superior/inferior views of a potted iliac wing illustrating the relationship between the wing and potting cup coordinate systems. The Notch to Belt Angle (green) is shown in c) as the difference between the Notch angle (purple) and the belt/loading angle (black). The Notch to Belt Angle was adjusted by rotating the iliac wing and potting cup on the test fixture within the plane shown in a). Adjustment of the pelvis roll angle was accomplished by rotating the iliac wing relative to the potting cup during the potting process in the plane shown in b).

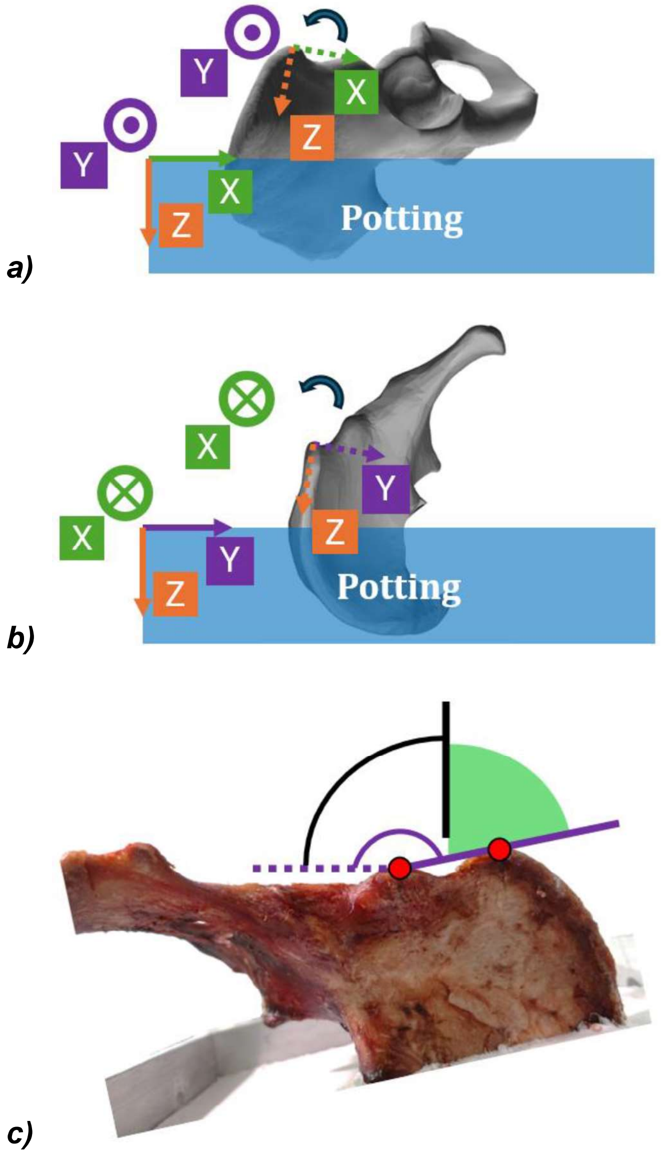

Figure A4: Photo of biaxial testing machine and test setup for component-level iliac wing fracture experiments.

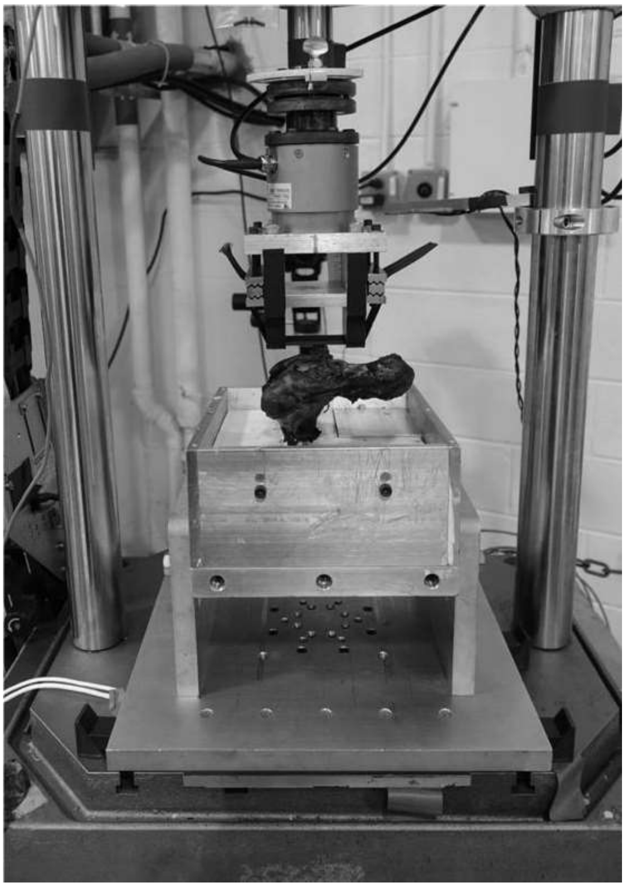

Table A1: Univariate Weibull survival model AICc values and p-values for each dataset (male, female, combined).

Model	AICc	p-value (covariate)
**Male**		
Base	328.6	[–]
BD	324.5	0.001*
Age	327.1	0.033*
Height	328.3	0.084
Weight	331.2	0.741
ASIS-PSIS	329.9	0.234
ASIS-PC	330.0	0.250
Angle	331.1	0.683
**Female**		
Base	274.9	[–]
Age	271.0	0.007*
Height	277.4	0.627
Weight	277.7	0.887
ASIS-PSIS	276.5	0.306
ASIS-PC	276.5	0.259
Position	277.2	0.506
**Combined**		
Base	598.8	[–]
Age	591.4	0.003*
Sex	600.8	0.567
Position	600.4	0.399
Angle	600.7	0.500

Figure A5**:** Example iliac wing injury from test (specimen 1078R).

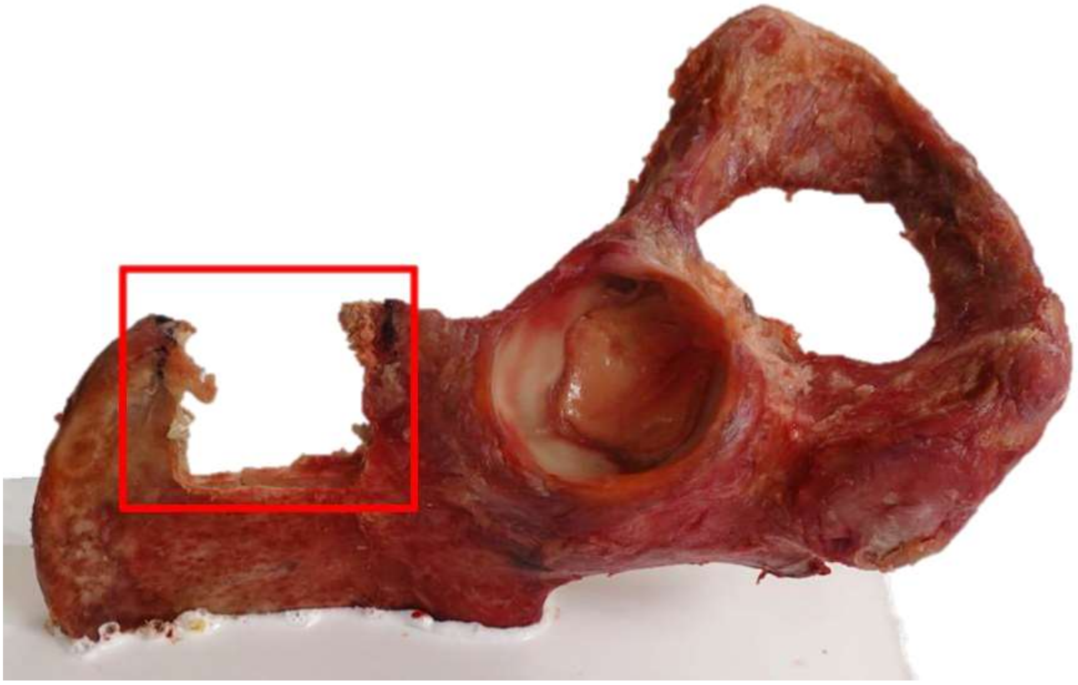

Figure A6: Correlation plot of fracture force for left and right sides of each female pelvis.

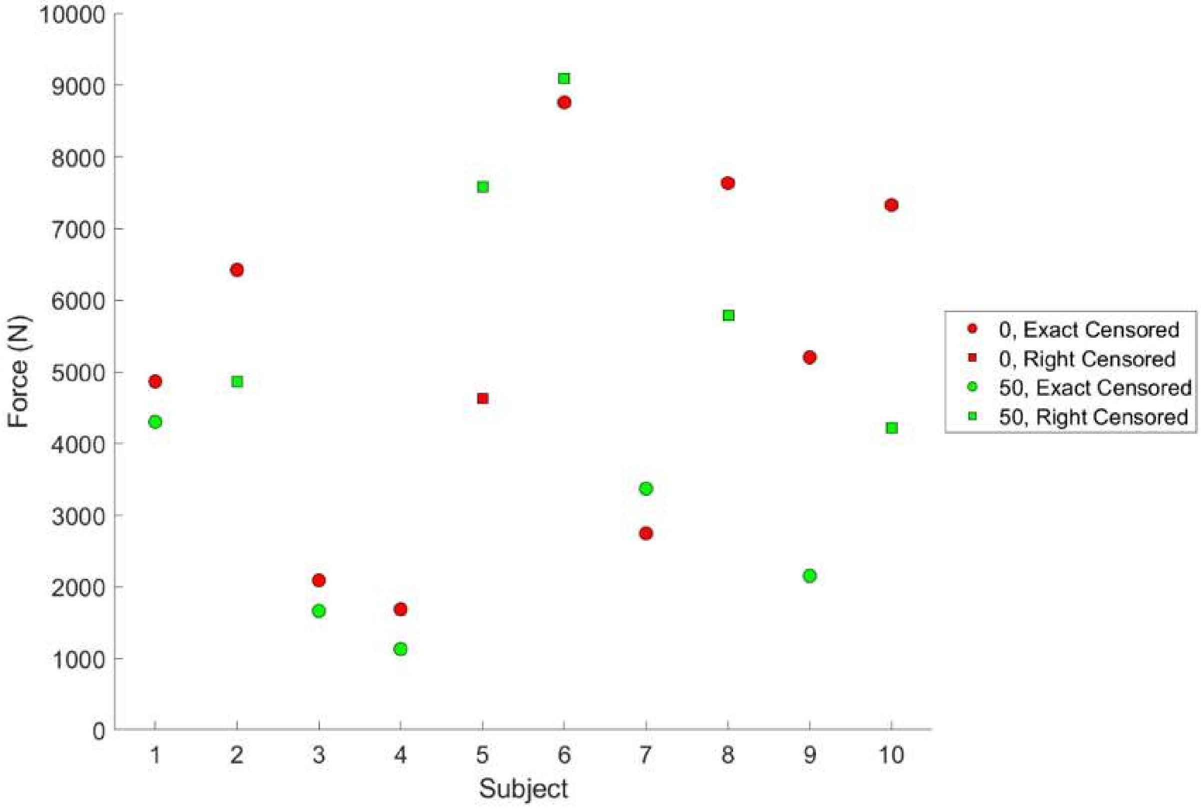

Supplement: Supplementary file 1 — Supplementary file1 (PDF 715 KB) [file 10439_2025_3777_MOESM1_ESM.pdf]
